# Effects of Protamine Reversal on Coagulation Parameters After High-Dose Heparin Administration in Percutaneous Hepatic Chemosaturation Intervention

**DOI:** 10.3390/clinpract15020038

**Published:** 2025-02-17

**Authors:** Michael Metze, Silke Zimmermann, Holger Kirsten, Robert Werdehausen, Rhea Veelken, Florian van Bömmel, Timm Denecke, Hans-Jonas Meyer, Sebastian Ebel, Manuel Florian Struck

**Affiliations:** 1 Center of Hemostaseology, Department of Cardiology, University Hospital Leipzig, Liebigstr. 20, 04103 Leipzig, Germany; michael.metze@medizin.uni-leipzig.de; 2 Institute of Laboratory Medicine, Clinical Chemistry and Molecular Diagnostics, University Hospital Leipzig, Paul-List-Str. 13/15, 04103 Leipzig, Germany; silke.zimmermann@medizin.uni-leipzig.de; 3 Institute for Medical Informatics, Statistics and Biometry, University of Leipzig, Härtelstr. 16-18, 04107 Leipzig, Germany; holger.kirsten@imise.uni-leipzig.de; 4 Department of Anesthesiology and Intensive Care Medicine, University Hospital Leipzig, Liebigstr. 20, 04103 Leipzig, Germany; robert.werdehausen@med.ovgu.de; 5 Department of Anesthesiology and Intensive Care Medicine, Medical Faculty, Magdeburg University, Leipziger Str. 44, 39120 Magdeburg, Germany; 6 Division of Hepatology, Department of Medicine II, University Hospital Leipzig, Liebigstr. 20, 04103 Leipzig, Germany; rhea.veelken@medizin.uni-leipzig.de (R.V.); florian.vanboemmel@medizin.uni-leipzig.de (F.v.B.); 7 Department of Diagnostic and Interventional Radiology, University Hospital Leipzig, Liebigstr. 20, 04103 Leipzig, Germany; timm.denecke@medizin.uni-leipzig.de (T.D.); hans-jonas.meyer@medizin.uni-leipzig.de (H.-J.M.); sebastian.ebel@medizin.uni-leipzig.de (S.E.)

**Keywords:** heparin effect, anticoagulation, protamine, heparin reversal, prothrombin, fibrinogen

## Abstract

Background: Intravenous protamine administration for heparin reversal after percutaneous hepatic chemosaturation intervention is generally recommended, but its effectiveness on coagulation parameters remains unclear. Methods: In a single-center retrospective observational study, the effects of different postinterventional protamine doses on the activated partial thromboplastin time (aPTT), international normalized ratio (INR), prothrombin time (PT), fibrinogen, platelet count (PLT), and hemoglobin (Hb) were analyzed in consecutive patients who underwent high-dose heparin administration (>300 U/kg body weight) and extracorporeal circulation for chemosaturation treatment. Due to the multiple treatments of individual patients, linear mixed-effects models were applied. Results: Thirty-one patients underwent 90 chemosaturation interventions, 68 (75.6%) of which involved heparin reversal with protamine. All investigated variables showed significant postinterventional alterations, while protamine use was associated with significantly lower aPTT, lower INR, higher PT, and higher fibrinogen levels, whereas PLT and Hb levels were comparable to those in procedures without protamine use. After adjustment for aPTT, significant independent effects of protamine remained for the INR and PT. Dose-dependent effects of protamine were observed for reductions in aPTT and an increase in fibrinogen levels, which were confirmed after adjustment for the heparin dose. A 10% higher protamine dose resulted in a 3% decrease in aPTT and a 4% increase in fibrinogen. An increase of 0.1 in the protamine-to-heparin ratio was associated with an increase of 9% in fibrinogen. Conclusions: The present results suggest that protamine contributes to the normalization of the aPTT, INR, PT, and fibrinogen levels. Further prospective studies should be conducted to determine optimal dosing ratios.

## 1. Introduction

Percutaneous hepatic melphalan perfusion (chemosaturation) has been a treatment option for primary and secondary liver tumors for more than 20 years [1]. The best treatment experience is in the hepatic metastases of the uveal melanoma, while patients with intrahepatic cholangiocarcinoma and other malignancies may also respond to this intervention [2,3,4,5]. Chemosaturation requires extracorporeal blood pump circulation and high-dose heparin anticoagulation to ensure circuit functionality and prevent the clotting of the filtration system [6,7]. Heparin-associated bleeding complications and prolonged coagulopathy can be avoided by the postinterventional administration of protamine [7]. Although the use of protamine is recommended by the manufacturer’s instructions, it may be associated with anaphylactic or thromboembolic complications and, at higher doses, paradoxical anticoagulant effects [7,8,9]. However, there is no clear recommendation as to whether it should be mandatory to reverse heparin with equivalent protamine dosages (with a 1:1 ratio of protamine to heparin) or whether it may be sufficient to provide reduced protamine doses. Data from cardiac surgery populations suggest that reduced protamine dosages are safe and may reduce potential protamine-related complications [10,11,12,13,14]. Activated partial thromboplastin time (aPTT) and activated clotting time (ACT) are the reference markers used to guide heparin treatment, while excessive dosages of heparin have known effects on other global coagulation parameters, including the international normalized ratio (INR) [15,16,17]. In a previous analysis, we observed that postoperative bleeding after hepatic chemosaturation is associated with the omission of protamine for heparin reversal [18]. Thus, the aim of this study was to determine the effect of protamine administration on prothrombin time (PT), INR, fibrinogen, platelet count (PLT), and hemoglobin (Hb) levels adjusted for aPTT, the heparin dose, and the protamine-to-heparin ratio.

## 2. Materials and Methods

This is a secondary analysis of a data set of consecutive patients who underwent percutaneous hepatic chemosaturation intervention at our institution between 2016 and 2022 [18]. The prerequisites for chemosaturation intervention in the study cohort were appropriate cerebrovascular condition, cardiopulmonary stability, and the absence of bleeding events (e.g., gastric ulcers, etc.). Patients with advanced liver cirrhosis (Child–Pugh score), pre-existing severe coagulation disorders, contrast agent allergies, or the presence of extrahepatic metastases, especially cerebral metastases, and lesions, were not suitable for this intervention due to their elevated risk for experiencing serious adverse events. These include thromboembolic complications, bleeding, and anaphylaxis [7].

In this study, coagulation variables with respect to postinterventional heparin reversal with protamine were analyzed. Ethical approval was obtained from the ethics committee at the Medical Faculty, Leipzig University, Leipzig, Germany (IRB00001750, project ID 500/20ek, 14 September 2020), and the need for informed consent was waived.

Patients scheduled for chemosaturation intervention underwent the placement of vascular catheters in the angiography unit following the induction of general anesthesia. After a bolus administration of heparin (300–400 U/kg), repeated ACT measurements were used to achieve target ACT levels >400 s, with further heparin titrations as needed. Extracorporeal circulation started after the blockade of the double-balloon catheter in the inferior cava vein. The intervention commenced by administering melphalan into the hepatic artery with the activation of the chemofilters of the bypass circuit. After the intervention, which took 2–3 h on average (including 1.5 h of extracorporeal perfusion time), a washout phase of 30 min was performed until the termination of extracorporeal circulation. Reversal of the heparin effect with protamine was performed in the angiography unit at the discretion of the treatment team after the discontinuation of the bypass circuit. All postinterventional samples (including those collected after protamine administration) were provided within the first 15 to 30 min after admission to the ICU. The interval between protamine administration and blood sample collection ranged from 30 to 60 min. The interval between heparin administration and protamine administration was consistent throughout the observation period, with a median extracorporeal circulation pump time of 92 min (interquartile range [IQR] 78–115.5 min). Postoperative management included monitoring in the intensive care unit for at least one night until the vascular catheters were removed [18].

The parameters studied were the heparin dose (total U and U/kg), protamine use (yes/no) and dose (mg), the protamine-to-heparin ratio, aPTT (s), PT (s), INR, fibrinogen (mg dL), PLT (10^9^/L), and Hb (mmol/L) levels. For the analysis of fibrinogen, the Clauss method is applied to an ACL Top analytics system (Fa. Werfen, HemosIL reagents, Munich, Germany). The fibrinogen concentration is measured via coagulometry. The tests for PT/INR use the reagent RecombiPlasTin (calcium and thromboplastin, HemosIL) and aPTT HemosIL SynthASil (Silica and calcium, Fa. Werfen, Munich, Germany) via coagulometry. All methods were stable regarding heparin concentrations of up to 1 U/mL and were measured in citrate plasma (1:9).

Hemoglobin concentration and platelet count are measured on the XN-9100 analytics system (Fa. Sysmex, Norderstedt, Germany) in EDTA blood. Platelet count is determined via hydrodynamic focusing and impedance measurements. Hemoglobin is determined via the SLS (sodium lauryl sulfate) hemoglobin method (cyanide-free).

The data were analyzed using R 4.3.2 (R Foundation for Statistical Computing, Vienna, Austria). Linear mixed-effects models were chosen to account for the non-independence of repeated procedures within subjects while allowing the estimation of both between- and within-subject variability in our unbalanced design. Outcome variables were transformed as needed to better fit the model assumptions. The R package rmcorr 0.6.0 was used to calculate the correlations between predictors, taking into account repeated measures. Mixed-effects linear regression was performed using the framework of generalized linear mixed models with transformed outcome variables, predictors, and a random intercept term representing the individual patient by applying the R package lme4 1.1.35. The natural logarithm (log) was used to transform aPTT, PLT, fibrinogen, and INR, while PT was squared. Confidence intervals were calculated by applying the Wald method as implemented in the package parameter 0.21.3. A *p*-value of 0.05 was considered to indicate statistical significance.

## 3. Results

The study cohort consisted of 31 patients who underwent 90 percutaneous hepatic chemosaturation procedures (cases). The majority of patients were female (n = 21; 67.7%) with a median (IQR) age of 61 (56–70) years. Uveal melanoma was the most frequent cancer disease in the study cohort (n = 17; 54.8%), followed by intrahepatic cholangiocarcinoma (n = 8; 25.8%), hepatocellular carcinoma (n = 2; 6.5%), and other types of carcinomas (n = 4; 12.9%). Twenty-one patients underwent repeated procedures ranging from two to six, while ten patients underwent only one procedure.

The median heparin dose was 30,000 U (median 429 U/kg). The protamine for heparin reversal was administered in 68 cases (75.8%), and the ratios of protamine (mg) to heparin (U) were 1:1 in 15 cases, 0.9:1 in 10 cases, 0.8:1 in 18 cases, 0.7:1 in 5 cases, 0.6:1 in 6 cases, 0.5:1 in 3 cases, 0.4:1 in 8 cases, and 0.3:1 in 3 cases. No protamine was used in 22 cases (24.2%). Postinterventional bleeding complications occurred in 13 cases (14.4%) (4 cases with protamine administration and 9 cases without), while no thromboembolic events or fatalities were observed during the study period [18]. The protamine-to-heparin ratios in the four cases of bleeding complications were 1:1 (twice), 0.9:1, and 0.75:1, respectively.

**Figure 1:**
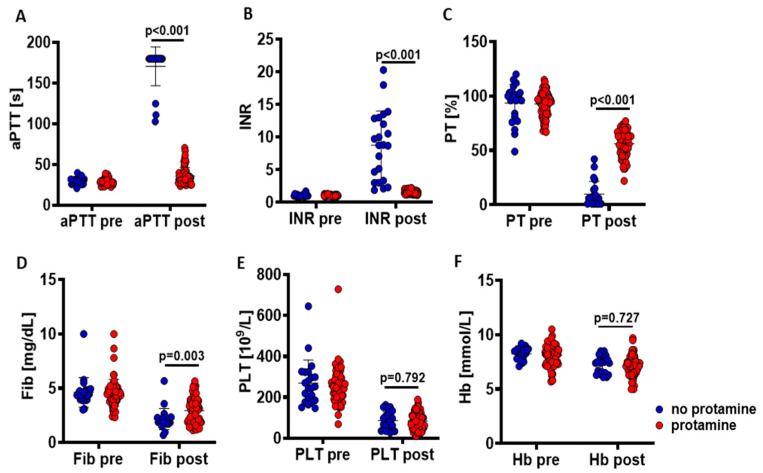
Outcome levels before and after percutaneous hepatic chemosaturation intervention with (red) and without (blue) heparin reversal with protamine. The baseline values of all variables (before chemosaturations) revealed no statistically significant differences in cases receiving protamine compared to those that did not (*p*-values > 0.35). Detailed statistics of postinterventional variables with respect to protamine administration are presented in Table 2. (**A**) aPTT, activated partial thromboplastin time; (**B**) INR, international normalized ratio; (**C**) PT, prothrombin time; (**D**) Fib, fibrinogen; (**E**) PLT, platelet count; (**F**) Hb, hemoglobin.

**Figure 2:**
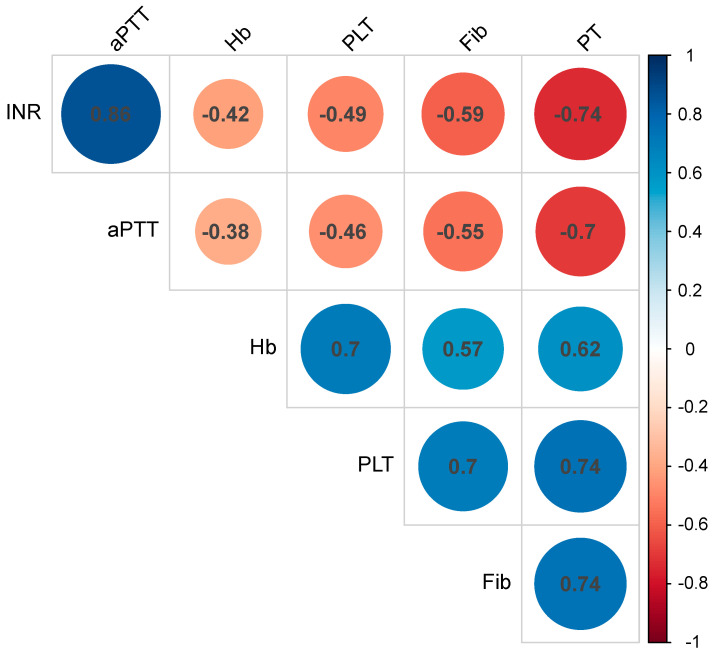
Correlations between investigated predictors, taking into account repeated measures. The size and color of the circles indicate the strength of the correlation. aPTT, activated partial thromboplastin time; INR, international normalized ratio; PT, prothrombin time; Fib, fibrinogen; PLT, platelet count; Hb, hemoglobin.

After the transformation of the outcome variables to apply for mixed-model analysis, significant correlations of coagulation parameters were observed (Figure 1), while the baseline levels were comparable between cases that received protamine and those cases that did not (aPTT, *p* = 0.647 (log-transformed); INR, *p* = 0.432 (log-transformed); PT, *p* = 684 (squared transformed); fibrinogen, *p* = 0.394 (log-transformed); PLT, *p* = 0.632 (log-transformed); Hb, *p* = 0.911 (not transformed)) (Figure 2).

All variables were significantly affected by the chemosaturation procedure itself (Table 1).

**Table 1:** Effects of percutaneous hepatic chemosaturation intervention on transformed outcomes.

Outcome	Coefficient (SE)	Explained Variance Intervention	Explained Variance Patient	*p*-Value
aPTT ^a^	1.7 (0.026)	96.6%	1.4%	<0.001
INR ^a^	1.9 (0.078)	84.5%	1.9%	<0.001
PT ^b^	−8900 (420)	73.4%	12.2%	<0.001
Fib ^a^	−0.78 (0.056)	53.4%	22.4%	<0.001
PLT ^a^	−1.2 (0.085)	57.5%	15.6%	<0.001
Hb ^c^	−0.99 (0.16)	16.2%	49%	<0.001

aPTT, activated partial thromboplastin time; INR, international normalized ratio; PT, prothrombin time; Fib, fibrinogen; PLT, platelet count; Hb, hemoglobin; ^a^ log transformation; ^b^ squared transformation; ^c^ no transformation; SE, standard error; CI, confidence interval.

Protamine administration had significant effects on the recovery of all coagulation parameters, whereas the Hb and PLT levels were comparable to those effects in cases without protamine (Figure 2, Table 2). After adjustment for aPTT, significant independent effects remained for the INR and PT, whereas the fibrinogen levels were comparable (Table 3).

**Table 2:** Effects of protamine administration on transformed outcomes.

Outcome	Coefficient (SE)	Explained Variance Protamine Administration	Explained Variance Patient	*p*-Value
aPTT ^a^	−1.6 (0.062)	88.9%	2%	<0.001
INR ^a^	−1.7 (0.099)	77.6%	6.1%	<0.001
PT ^b^	3300 (290)	58.3%	18%	<0.001
Fib ^a^	0.31 (0.1)	9.4%	36.6%	0.003
PLT ^a^	−0.038 (0.15)	0.1%	2.6%	0.792
Hb ^c^	0.082 (0.23)	0.1%	41.6%	0.727

aPTT, activated partial thromboplastin time; INR, international normalized ratio; PT, prothrombin time; Fib, fibrinogen; PLT, platelet count; Hb, hemoglobin; ^a^ log transformation; ^b^ squared transformation; ^c^ no transformation; SE, standard error; CI, confidence interval.

The dose-dependent effects of protamine use were assessed by including the significant variables aPTT, INR, PT, and fibrinogen in the analysis. Thereby, the protamine dose (in mg) was log-transformed. Taking into account the double log transformation of predictors and outcomes, the data suggest that a 10% increase in the protamine dose resulted in an approximately 3% decrease in the aPTT and a 4% increase in the fibrinogen level (Table 4). Analyzing the dose dependency of protamine adjusted for the heparin dose revealed similar results for aPTT and fibrinogen, with stronger effects when using heparin in units per kilogram of body weight rather than the total units of heparin (Table 5 and Table 6).

When the protamine-to-heparin ratio (non-log transformed) was analyzed to predict the outcome of coagulation parameters, an increase of 0.1 in the protamine-to-heparin ratio was associated with an increase of approximately 9% for the fibrinogen level (Table 7).

The relevance of the protamine dosage and the protamine-to-heparin ratio on fibrinogen levels were similar because they both accounted for approximately 11% of the variance in fibrinogen.

## 4. Discussion

The present results confirm previous reports that percutaneous hepatic chemosaturation, including high-dose heparin administration, extracorporeal circulation, and chemofilter application, significantly increases aPTT and INR and decreases PT, fibrinogen, PLT, and Hb levels [1,2,3,4,5,6,8]. The novel findings are that heparin reversal with protamine not only normalizes aPTT but also restores the INR, PT, and fibrinogen levels, which are effects that remained stable for the INR and PT after adjustment for aPTT. Furthermore, a dose-dependent association between the aPTT and fibrinogen level was observed for protamine.

Although similar effects were observed in patients who underwent cardiopulmonary bypass during cardiac surgery [19], the specific interactions of heparin and protamine with the INR, PT, and fibrinogen have not been demonstrated in cohorts of patients with hepatic chemosaturation.

### 4.1. Characteristics of Heparin Effects

The effect of heparin on PT may be the result of the binding of heparin to antithrombin, which causes a conformational change that leads to its activation. Antithrombin then exerts its inactivating effects primarily on thrombin and factor Xa. Since thrombin (which is involved in the common coagulation pathway of the coagulation cascade) is inhibited, it prolongs PT. However, two measures are responsible for allowing the measurement of PT in the presence of therapeutic heparin: first, the dilution factor, and second, heparin neutralizers (e.g., heparinase), which are used in most commercial PT assays [20]. Since heparin in blood samples influences the performance of PT reagents, PT reagents may compensate for 1–2 U/mL of heparin, whereas excess dosages ultimately still lead to prolonged PT and INR [21,22]. The different sensitivities of PT measurements when using different reagent charges may also influence the interpretation of the results. The high dependency of the results on different commercial assays was the historical reason for the development of the INR to improve comparability [23].

Fibrinogen levels after hepatic chemosaturation without heparin reversal with protamine levels were significantly lower than those with protamine (Figure 1D), which would be presumably affected by high doses of heparin in the measurement of fibrinogen, as reported in the cardio-pulmonary bypass [19]. Those pseudo-low levels of fibrin during measurement probably reflect the results of Table 2, Table 3, Table 4, Table 5, Table 6 and Table 7.

### 4.2. Characteristics of Protamine Effects

Heparin reversal with protamine is considered mandatory in percutaneous hepatic chemosaturation procedures [7]. The normalization of coagulation parameters, usually observed within 24 h after the procedure, is important before the removal of the vascular sheaths. Prolonged coagulopathy can lead to severe bleeding complications, which may include airway compromise, stridor, and impaired laryngoscopy visibility if emergency tracheal intubation is required [6]. This may be particularly true after the removal of the large bore sheath and central venous catheter from the internal jugular vein. In addition, angiographic bleeding control or open surgical repair has been reported in the postoperative period following hepatic chemosaturation. However, few studies have specifically analyzed postoperative coagulation management in chemosaturation patients with or without protamine administration [9,18]. After one center abandoned postinterventional protamine reversal due to severe thromboembolic complications (two cases of cerebral ischemia in 141 procedures in 60 patients), our center also decided to use protamine in a more individualized approach and case-by-case decision [8]. However, we changed our standards back to the mandatory use of protamine after experiencing several severe bleeding complications. Another recent two-center study involving 256 procedures in 116 patients (including the data sets of the previously cited study from Hannover, Germany, and another center from Hamburg, Germany) suggested that the standard use of protamine after chemosaturation in low-risk patients without clinical signs of active bleeding should be critically re-evaluated [9]. In this population, ten thromboembolic events (seven ischemic strokes and one each of myocardial infarction, deep vein thrombosis, and pulmonary embolism) were found in 192 cases of 92 patients who received full protamine reversal (1:1 ratio), compared with no thromboembolic events in the reduced protamine reversal (21 cases of 13 patients) and no-reversal (43 cases of 28 patients) groups. Regarding bleeding complications, 24 events were observed in the full reversal group, two in the reduced reversal group, and 12 in the no-reversal group. ACT rates were significantly higher in the no reversal group at the last measurement after chemosaturation. Interestingly, the highest rate of thrombocytopenia was observed in the full reversal group (39%) compared to reduced and no reversal (14% and 23%, respectively). However, thrombocytopenia was only classified as binary, and a platelet count was not presented, and details on other coagulation parameters (INR, PT, aPTT, and fibrinogen) were not presented. In addition, one case of severe anaphylactic shock was observed after protamine administration. In the current data set, an independent protective effect of protamine against postoperative bleeding complications was observed, while thromboembolic events were not observed, presumably due to the high proportion of cases receiving protamine-to-heparin ratios of less than 1:1 [18].

The overall efficacy of protamine reversal has been extensively studied. In a recent randomized controlled trial of patients undergoing transfemoral transcatheter aortic valve implantation, the routine administration of protamine resulted in a reduction in minor vascular complications, procedure time, and post-procedural hospital stay compared to patients receiving a placebo [24]. The safety profile of protamine in study cohorts of different interventions suggests that the rate of adverse events (e.g., hypersensitivity reaction, stent thrombosis, myocardial infarction, pulmonary embolism, and stroke) is low [25,26,27]. Therefore, a reduced dose regime of protamine might be a promising strategy to reduce or avoid adverse events. This finding is supported by recent studies in cardiac surgery patients showing that reducing the protamine dose did not significantly increase blood loss [25,28]. Clinical concerns include the possibility of protamine overdose and subsequent paradoxical bleeding [10,11]. Protamine may reduce both platelet count and platelet function, probably by impairing the interaction of glycoprotein iB with the von Willebrand factor [29]. Therefore, the complex interactions of heparin and protamine have a relevant influence on clinical decisions [30,31].

Determining an optimal protamine-to-heparin ratio for all patients poses methodological challenges. First, heparin response varies widely among patients due to differences in body weight, age, renal function, and individual sensitivity, which complicates the determination of a universal protamine-to-heparin ratio that is effective for all patients [32]. Secondly, conventional assays, such as activated partial thromboplastin time (aPTT), lack the requisite sensitivity to accurately reflect heparin concentration due to their inherent variability. On the other hand, more specifically, anti-factor Xa assays are neither always available nor validated for protamine adjustments [33]. Thirdly, protamine may lead to impaired clot formation and inhibit thrombin formation if over-administered when given in ratios exceeding 1:1 [32]. Finally, protamine and heparin differ in their pharmacokinetic profiles. While protamine demonstrates rapid action and a brief duration of action, heparin can exhibit prolonged activity. The future may entail the implementation of thrombin generation assays; however, these are not yet part of standard clinical practice.

The present findings suggest that the normalization of the INR, PT, and fibrinogen should be confirmed after hepatic chemosaturation even when immediate postinterventional ACT and aPTT measurements reveal normal values. Furthermore, vascular sheaths should remain in place until approximately 12 h after the intervention, and the puncture sites should be monitored closely to avoid bleeding complications.

In general, protamine should be infused slowly and with extreme caution. Its potential to cause severe anaphylactic reactions, including shock and cardiac arrest, is based on the release of histamine from tissue mast cells within the blood vessels. Emergency treatment includes the rapid infusion of large volumes of crystalloid and vasopressors (epinephrine) [34]. An individual predisposition to allergic reactions to protamine can be screened during the initial selection of patients.

### 4.3. Limitations

The main limitations of this analysis are the retrospective approach, the single-center design, and the limited number of observations. The retrospective design carries the risk of documentation issues and is inferior to prospectively collected data that are designed for a specific research purpose. The monocentric nature further limits the generalizability of the results, and selection and recall bias should be considered. Although we used a mixed-model approach for repeated measures and random intercepts, confounding effects cannot be excluded due to the small sample size. Due to the low and inconsistent frequency of the different protamine-to-heparin ratios used, it is not possible to recommend safe threshold ratios based on the current data set. Further prospective studies are needed to determine optimal dosing ratios. However, the number of procedures included in this analysis renders it the most comprehensive collection of data on this topic to date. Furthermore, heparin reversal was performed using protamine under the discretion of the attending team and was not applied due to a study protocol. We present new data generated under conditions of real-world treatments. Finally, although this study provides valuable insights into the coagulation profile and its association with clinical outcomes, it is important to acknowledge that our analysis focused primarily on conventional coagulation parameters such as aPTT, INR, PT, and platelet count. While these parameters are routinely used to assess coagulation status and bleeding risk, they may not fully capture the complexity of hemostasis or account for individual variability. Emerging evidence suggests that additional factors may play a critical role in coagulation and bleeding risk, including levels of individual coagulation factors, the presence of specific antibodies, and alterations in fibrinolytic pathways and thrombin generation [10,35]. In addition, structural and functional abnormalities of platelets or endothelial cells, as well as genetic or acquired defects in coagulation pathways, may also significantly contribute to the risk of bleeding or thrombotic events. The omission of these parameters from our study may limit the comprehensiveness of our findings. Future studies incorporating a broader range of hemostatic markers and a more personalized approach to coagulation assessment will be essential to deepen our understanding of bleeding risk and refine clinical decision making.

## 5. Conclusions

Percutaneous hepatic chemosaturation procedures, including high-dose heparin administration, were associated with alterations in the aPTT, INR, PT, fibrinogen, PLT, and Hb. Postinterventional protamine administration had restorative effects on aPTT, PT, INR, and fibrinogen levels but not on Hb or PLT levels. The effects of protamine on the INR and PT levels appeared to be independent of the aPTT and dose-dependent for its effects on the aPTT and fibrinogen levels. Further prospective studies should be conducted to determine optimal dosing ratios. These should include appropriate sample sizes in fixed groups of different protamine-to-heparin ratios (e.g., 0.75:1, 0.5:1, and 0.25:1, or 0.66:1 and 0.33:1) to assess the minimum dose required to avoid bleeding complications and potential adverse effects. The effect of protamine should also be studied in relation to different tumor types and include a broader range of hemostatic markers.

## Figures and Tables

**Table 3:** Effects of protamine administration adjusted to activated partial thromboplastin time.

Outcome	Coefficient (SE)	*p*-Value
INR ^a^	−1.9 (0.28)	<0.001
PT ^b^	3900 (800)	<0.001
Fib ^a^	0.41 (0.28)	0.151
PLT ^a^	0.29 (0.44)	0.517
Hb ^c^	0.55 (0.64)	0.392

INR, international normalized ratio; PT, prothrombin time; Fib, fibrinogen; PLT, platelet count; Hb, hemoglobin; ^a^ log transformation; ^b^ squared transformation; ^c^ no transformation; SE, standard error; CI, confidence interval.

**Table 4:** Dose-dependent effects of protamine on transformed outcomes.

Outcome	Coefficient (SE)	Outcome % Change for 10% Protamine Change (95% CI)	Explained Variance Protamine	Explained Variance Patient	*p*-Value
aPTT ^a^	−0.299 (0.078)	−2.8% (−4.2–−1.3%)	18%	0%	<0.001
INR ^a^	0.043 (0.056)	n.s.	0.9%	57.4%	0.443
PT ^b^	−195 (472)	n.s.	0.3%	48.9%	0.681
Fib ^a^	0.369 (0.137)	3.5% (0.9–6.3%)	11.1%	20.8%	0.009

aPTT, activated partial thromboplastin time; INR, international normalized ratio; PT, prothrombin time; Fib, fibrinogen; ^a^ log transformation; ^b^ squared transformation; SE, standard error; CI, confidence interval. n.s.; not statistically significant.

**Table 5:** Dose-dependent effects of protamine adjusted to absolute heparin dose (total U).

Outcome	Coefficient (SE)	*p*-Value
aPTT ^a^	−0.25 (0.082)	0.004
INR ^a^	0.057 (0.059)	0.334
PT ^b^	−360 (490)	0.468
Fib ^a^	0.39 (0.15)	0.01

aPTT, activated partial thromboplastin time; INR, international normalized ratio; PT, prothrombin time; Fib, fibrinogen; ^a^ log transformation; ^b^ squared transformation; SE, standard error.

**Table 6:** Dose-dependent effects of protamine adjusted to weight-related heparin dose (U per kg body weight).

Outcome	Coefficient (SE)	*p*-Value
aPTT ^a^	−0.28 (0.084)	0.001
INR ^a^	0.049 (0.061)	0.423
PT ^b^	−320 (510)	0.532
Fib ^a^	0.49 (0.14)	0.001

aPTT, activated partial thromboplastin time; INR, international normalized ratio; PT, prothrombin time; Fib, fibrinogen; ^a^ log transformation; ^b^ squared transformation; SE, standard error.

**Table 7:** Effects of protamine-to-heparin ratio on transformed outcomes.

Outcome	Coefficient (SE)	Outcome % Change for 0.1 Unit Protamine-to-Heparin Ratio Change (95% CI)	Explained Variance Protamine-to-Heparin Ratio	Explained Variance Patient	*p*-Value
aPTT ^a^	−0.19 (0.15)	n.s.	2.6%	19.4%	0.209
INR ^a^	0.079 (0.089)	n.s.	1.1%	54.5%	0.382
PT ^b^	−761 (753)	n.s.	1.5%	48.1%	0.316
Fib ^a^	0.618 (0.226)	8.5% (1.8–19.1%)	11%	24.7%	0.008

aPTT, activated partial thromboplastin time; INR, international normalized ratio; PT, prothrombin time; Fib, fibrinogen; ^a^ log transformation; ^b^ squared transformation; SE, standard error; CI, confidence interval. n.s. not statistically significant.

## Data Availability

The data set supporting the conclusions of this article is available from the corresponding author upon reasonable request.
